# Vaccination for Mpox (Monkeypox) Infection in Humans: From Basic Science to Real-World Effectiveness

**DOI:** 10.3390/vaccines12101147

**Published:** 2024-10-08

**Authors:** Kay Choong See

**Affiliations:** Division of Respiratory and Critical Care Medicine, Department of Medicine, National University Hospital, Singapore 119074, Singapore; kaychoongsee@nus.edu.sg

Human mpox (previously known as monkeypox) is a multi-system disease caused by an orthopox DNA virus [1]. Spread can be rapid, predominantly via close contact and possibly via respiratory secretions. Within dense networks, such as men who have sex with men (MSM) communities, without vaccination, 68.2–89.7% of individuals will be infected with mpox over one year [2]. Due to the significant and rapid global spread of mpox, primarily driven by a Clade II outbreak, the World Health Organization (WHO) declared mpox a Public Health Emergency of International Concern (PHEIC) on 23 July 2022 [3]. After a sustained decline in global cases, the WHO lifted the PHEIC status on 11 May 2023 [4]. However, on 14 August 2024, the WHO once again declared mpox a PHEIC following a resurgence, this time primarily involving a Clade I outbreak [5]. Between January 2022 and August 2024, more than 120 countries have reported over 100,000 laboratory-confirmed cases of human mpox [6]. Although mpox usually runs a benign course, deaths may ensue [1]. Among the laboratory-confirmed cases recorded by WHO, more than 200 deaths resulted [6]. The case fatality rate was about 11% among children, and about 50% of maternal infections during pregnancy led to fetal loss [7].

The highly pathogenic variola virus causes smallpox, while the closely related vaccinia virus is used in vaccines to provide immune protection against it. Apart from smallpox, the antibodies induced by the vaccinia vaccine are cross-protective for other orthopoxviruses, such as mpox. First- and second-generation smallpox vaccines contain live, unattenuated vaccinia virus and can cause serious side effects in a small percentage of recipients, with death occurring in 1 to 10 cases per million vaccinations. In contrast, third-generation vaccines, which use attenuated vaccinia strains, are much safer, produce milder side effects, and can be given to pregnant women, children, and immunocompromised patients. LC16m8, which is a replicating attenuated strain of vaccinia, seems to have mild side effects like third-generation smallpox vaccines [8]. Other vaccine alternatives in development include a recombinant chimeric horsepox virus TNX-801 vaccine [9], a subunit Ad35 vector-based vaccine [10], a non-replicating vaccinia virus vaccine derived from the Tian Tan strain [11], a polyvalent mRNA-based vaccina virus vaccine [12], an mRNA-lipid nanoparticle vaccine expressing mpox virus surface proteins [13], and a multivalent mRNA orthopoxvirus vaccine encoding mpox virus antigens [14].

Vaccination is an important strategy for mitigating the mpox outbreak, but contemporary data on its efficacy, utility, and adverse effects are needed. In this Special Issue—Vaccination for Monkeypox Infection in Humans—a series of papers covering the basic and clinical sciences outlined contemporary knowledge on mpox. The series started with a review of the key considerations for vaccination and highlighted the need to vaccinate healthcare workers at elevated risk for occupational exposure, immunocompromised patients, and children (https://doi.org/10.3390/vaccines10081342). In addition, vaccination strategies included pre-exposure vaccination, post-exposure prophylaxis, and ring vaccination of close contacts. Malik and colleagues then extended our understanding of mpox by providing two comprehensive reviews of viral pathology, immune response, contemporary vaccines, and antiviral agents (https://doi.org/10.3390/vaccines11030500; https://doi.org/10.3390/vaccines11081345). Despite the availability of vaccines, awareness of mpox can be a barrier to vaccination success. Sahin and colleagues performed a survey of 283 Turkish physicians in August-September 2022, which was after the first PHEIC declaration, and showed that only 32.5% of physicians were familiar with mpox and only 31.4% planned to be vaccinated (https://doi.org/10.3390/vaccines11010019).

Vaccine efficacy measures how well a vaccine reduces specific outcomes in controlled settings like randomized clinical trials. In contrast, vaccine effectiveness refers to how well the vaccine performs in real-world conditions. Historical smallpox vaccination has a vaccine effectiveness of between 40 and 80% against mpox Clade II, though information for Clade I is unavailable [15]. Two other papers showed that currently available vaccines are immunogenic and safe, but also revealed gaps in knowledge among severely immunocompromised patients, and in real-world settings. Among 33 HIV-positive patients, all of whom had CD4+ T cell counts of 335/µL and above, Stefanie and colleagues demonstrated that vaccination against smallpox and mpox successfully generated T cell responses against these viruses (https://doi.org/10.3390/vaccines12020131). Their results support the efficacy of mpox vaccination in HIV-positive patients, though these patients should not be severely immunocompromised. In their systematic review and meta-analysis, Nave and colleagues showed that the Modified Vaccinia Ankara (MVA) vaccine for mpox generated protective antibodies after either one or two doses and did not have any severe cardiovascular adverse events (https://doi.org/10.3390/vaccines11091410). They also showed that existing studies until 28 June 2023 had data on vaccine efficacy but lacked data on clinical effectiveness.

Other published studies have partly filled the gaps in knowledge identified in this Special Issue. These studies have not only shown that first- to third-generation smallpox vaccines are effective in the real world [16,17,18,19,20,21,22,23,24,25] and in immunocompromised individuals [19], but they have also suggested dose-conservation regimes to extend limited vaccine supplies (see Table 1). For instance, single-dose vaccination with the third-generation MVA-Bavarian Nordic vaccine has been used to optimize limited vaccine supplies. The results have been promising, showing an effectiveness of about ten percentage points lower than the full two-dose regimen [17,18,19,20,21]. Additionally, breakthrough cases of mpox following single-dose vaccination tend to present with mild clinical symptoms [26].

In conclusion, the papers in this Special Issue provide fair support for mpox vaccination to prevent disease and preserve health. Nonetheless, challenges to successful vaccination remain, including a need to overcome mpox vaccine hesitancy. A systematic review of studies focusing on knowledge, attitudes, and perceptions showed that 53.4% of participants had poor knowledge of mpox [27]. Furthermore, global vaccine acceptance and uptake rates were 59.7% and 30.9% respectively [28]. Related to vaccine hesitancy is the need to convince individuals that vaccine effectiveness is long-lasting, but the data remain lacking. Conversely, a study from the Netherlands calls to question the durability of protection afforded by mpox vaccination in people without prior vaccination against smallpox or mpox. Van Leeuwen and colleagues showed that orthopoxvirus-specific binding and MVA-neutralizing antibodies declined to undetectable levels within one year after vaccination in at-risk individuals who had completed the full two-dose course of the MVA-Bavarian Nordic vaccine [29]. Furthermore, current studies assume vaccine efficacy and effectiveness against Clade II would extend to Clade I mpox infections. While this assumption is reasonable, mpox vaccines should also be studied for Clade I infections to ensure their effectiveness [13,14], given the greater virulence of Clade I as opposed to Clade II.

## Figures and Tables

**Table 1:** Effectiveness of mpox vaccines that have been used in clinical settings.

Vaccine Name	Vaccine Type	Dosing Method	Effectiveness
Dryvax (Wyeth Laboratories, Madison, NJ, USA)	First generation smallpox vaccine with live replicating vaccinia virus	One dose via skin-prick using a bifurcated stainless-steel needle	72% [16]
ACAM2000 (Emergent Biosolutions, Gaithersburg, Maryland, USA)	Second generation smallpox vaccine with live replicating vaccinia virus	One dose via skin-prick using a bifurcated stainless-steel needle	75% [16]
Modified Vaccinia Ankara-Bavarian Nordic (MVA-BN) (Bavarian Nordic, Hellerup, Denmark) (Imvamune^TM^, Imvanex^TM^, Jynneos^TM^)	Third generation smallpox vaccine with live attenuated, nonreplicating vaccinia virus	One subcutaneous 0.5 mL dose	35–80% * [17,18,19,20,21,22,23,24,25]
		Two subcutaneous 0.5 mL doses, ≥28 days apart	66–89% [20,22,23,24]
		Two intradermal 0.1 mL doses, ≥28 days apart	85.9% [19] IC: 70.2% [19]
LC16m8 (KM Biologics, Kumamoto, Japan)	Replicating attenuated strain of vaccinia	One dose via skin-prick using a bifurcated stainless-steel needle	Data are not available

* At ≥14 days post-vaccination. IC: immunocompromised individuals.
